# Enhanced expression of G-protein coupled estrogen receptor (GPER/GPR30) in lung cancer

**DOI:** 10.1186/1471-2407-12-624

**Published:** 2012-12-28

**Authors:** Venkatakrishna Rao Jala, Brandie N Radde, Bodduluri Haribabu, Carolyn M Klinge

**Affiliations:** 1 James Graham Brown Cancer Center, Department of Microbiology and Immunology, 505 South Hancock Street, Room 323, CTR Building, Louisville, KY 40202, USA; 2 Department of Biochemistry and Molecular Biology, and Center for Genetics and Molecular Medicine, University of Louisville School of Medicine, Louisville, KY, 40202, USA

**Keywords:** GPER, GPR30, Estrogen, Estrogen receptor, Lung cancer, Protein expression, Immunohistochemistry, Tissue microarray

## Abstract

**Background:**

G-protein-coupled estrogen receptor (GPER/GPR30) was reported to bind 17β-estradiol (E_2_), tamoxifen, and ICI 182,780 (fulvestrant) and promotes activation of epidermal growth factor receptor (EGFR)-mediated signaling in breast, endometrial and thyroid cancer cells. Although lung adenocarcinomas express estrogen receptors α and β (ERα and ERβ), the expression of GPER in lung cancer has not been investigated. The purpose of this study was to examine the expression of GPER in lung cancer.

**Methods:**

The expression patterns of GPER in various lung cancer lines and lung tumors were investigated using standard quantitative real time PCR (at mRNA levels), Western blot and immunohistochemistry (IHC) methods (at protein levels). The expression of GPER was scored and the pairwise comparisons (cancer *vs* adjacent tissues as well as cancer *vs* normal lung tissues) were performed.

**Results:**

Analysis by real-time PCR and Western blotting revealed a significantly higher expression of GPER at both mRNA and protein levels in human non small cell lung cancer cell (NSCLC) lines relative to immortalized normal lung bronchial epithelial cells (HBECs). The virally immortalized human small airway epithelial cell line HPL1D showed higher expression than HBECs and similar expression to NSCLC cells. Immunohistochemical analysis of tissue sections of murine lung adenomas as well as human lung adenocarcinomas, squamous cell carcinomas and non-small cell lung carcinomas showed consistently higher expression of GPER in the tumor relative to the surrounding non-tumor tissue.

**Conclusion:**

The results from this study demonstrate increased GPER expression in lung cancer cells and tumors compared to normal lung. Further evaluation of the function and regulation of GPER will be necessary to determine if GPER is a marker of lung cancer progression.

## Background

Lung cancer is the leading cause of cancer deaths in United State of America both in men and women [1]. Although controversial, some epidemiologic data indicate that women have a higher risk of lung adenocarcinoma, a type of non-small cell lung cancer (NSCLC), compared to men, independent of smoking status [2,3]. One recent study reported reduced risk of lung cancer mortality in breast cancer patients, who were taking antiestrogens [4]. This study also found that women taking antiestrogens had a significant lower risk of developing lung cancer [4]. While it is known that estrogens induce maturation of normal lung tissue [5,6], their role in lung cancer initiation and progression remains unclear.

Estrogens regulate a wide variety of biological processes including differentiation, cell proliferation, apoptosis, inflammation and metabolism primarily by binding to two receptors: ERα and ERβ (ERs will refer to both subtypes) [7-12]. ERα and ERβ belong to the nuclear receptor superfamily of ligand-activated DNA binding transcription factors (reviewed in [13]). The classical mechanism of E_2_ action involves binding to ERs to form homo- or hetero- dimers followed by direct binding to estrogen response elements (ERE) or tethering to other DNA bound-transcription factors, *e.g*., AP-1, located in the regulatory regions of target genes [14]. The resulting recruitment of co-activators and chromatin remodeling complexes alters gene transcription leading to physiological responses within hours following E_2_ exposure. Estrogens also promote various types of cancers including breast cancer and ablation of estrogen synthesis or ER activities are effective treatments to prevent disease recurrence [15].

ERα and ERβ proteins are expressed in primary lung tumors (reviewed in [5]). In contrast to breast cancer, ERβ levels are ~ twice that of ERα levels in lung cancers [16,17]. It was also reported that NSCLC cells express ERα and ERβ and respond transcriptionally to E_2_[18-22]. In addition to the classical genomic mechanism of estrogen action, numerous studies have demonstrated that E_2_ rapidly (in < 5 min.) activates plasma membrane initiated signaling cascades through G-Protein dependent pathways, including release of intracellular calcium, IP3 accumulation, cAMP production, and MAPK activation [23-26]. Both ERα [27,28] and ERβ [29] appear to localize with protein kinases and other proteins in ‘signalosome’ complexes in caveolae in the plasma membrane in a cell type-dependent manner. In this context, the non-genomic E_2_-ERβ dependent signaling and cooperation between β1adrenergic receptor and ERβ signaling pathways may contribute to the smoking-associated lung carcinoma progression in women [30]. There is also considerable evidence for a role for E_2_ activation of membrane-associated ER crosstalk with epidermal growth factor receptor (EGFR) (reviewed in [10,31-38]).

GPR30/GPER (also known as DRY12, FEG-1, LERGU, LyGPR, CMKRL2, LERGU2 and GPCR-Br) was first identified as a GPCR involved in membrane-mediated E_2_- signaling [39-42]. The precise role of GPER, its intracellular location, and role in mediating estrogen function remains controversial [27,43-46]. GPER was reported to bind E_2_ with high affinity (Kd = 3–7 nM) and to activate multiple intracellular signal transduction pathways, *e.g*., calcium mobilization, cAMP production, PI3K activation and ERK1/2 activation in a G-protein dependent manner. Northern blot, real time PCR, and immunohistochemistry (IHC) analyses showed that GPER is expressed in placenta, heart, lung, liver, prostate, bone marrow and fetal liver [47], but a complete atlas of GPER protein expression and its functional roles are yet to be established**.** Here, we report for first time, the expression patterns of GPER in lung cancer cell lines and human lung cancer tissues. The results from our studies indicate that the expression of GPER is elevated in lung tumors compared to normal/adjacent lung tissues.

## Results

### Expression of GPER in lung cancer cell lines

**Figure 1:**
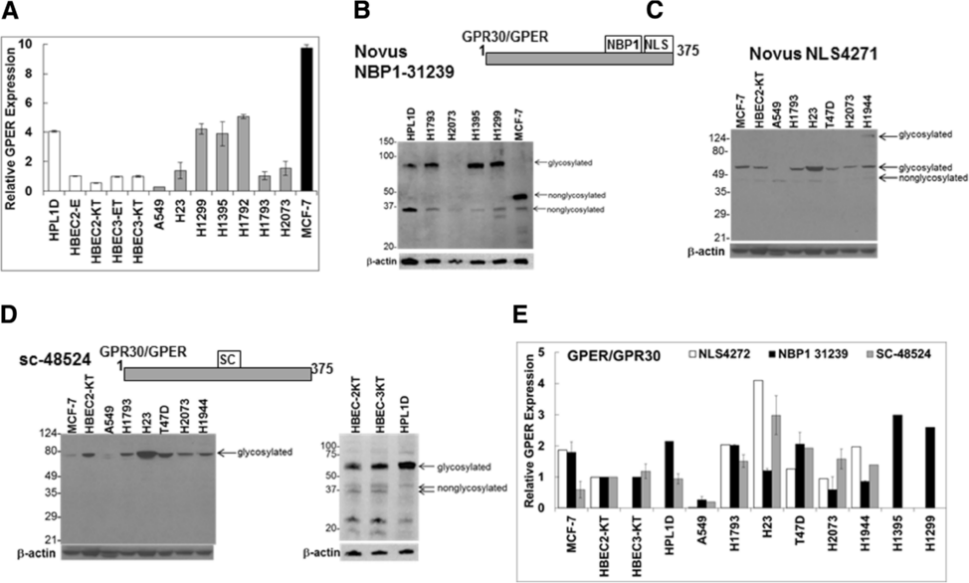
**GPER/GPR30 expression in normal lung cell lines and lung adenocarcinoma cell lines. A**) The expression of GPER was determined by realtime quantitative PCR in each of the normal lung cell lines (open bars), lung adenocarcinoma cell lines (grey bars), and MCF-7 human breast cancer cells (black bar). Values are the average of 4–6 biological replicates ± SEM. B-D) Representative western blot analysis of GPER/GPR30 expression in the indicted cell lines. 30 μg of whole cell extract (WCE) were separated on 10% SDS PAGE gels with MW marker migration indicated at the left of each blot. The diagrams at the top of B-C, and D indicate the region of GPER recognized by the 3 different polyclonal antibodies used: **B**) Novus NBP-1-31239, **C**) Novus NLS4271; **D**) Santa Cruz sc-4854-R. The MW of GPR30 is estimated to be 42 kDa, but higher MW sizes have been reported due to glycosylation and interaction with other proteins. Bands are identified as glycosylated and nonglycosylated based on reports cited in the text, but could include interaction with other proteins. For each GPER western, the membrane was stripped and reprobed for β-actin as a loading control. Quantitation of GPER was evaluated by summing all immunoreactive bands and dividing by β-actin, then normalizing to HBEC2-KT in each blot. Panel **E** is a summary of all westerns (not all westerns are shown) with each antibody. For Westerns with NBP1-31239 and sc-48524, values are the mean ± SEM from 3 separate blots using different WCE.

Although at the time of its cloning, GPER was shown to be expressed in normal human lung by Northern blot [48], its expression in lung cancer cell lines or lung tumors has not been examined. The mRNA levels of GPER were determined in human NSCLC, lung adenocarcinoma cell lines: A549, NCI-H23, NCI-H1299, NCI-H1792, NCI-H1395, NCI-H1435, NCI-H1793, NCI-H1944, NCI-H2073 (all purchased from ATCC); immortalized, but not transformed, human bronchial epithelial lung cell lines obtained from Dr. John D. Minna, UT Southwestern: HBEC3-KT, HBEC2-E and HBEC2-KT [49]; the SV40-immortalized human small airway epithelial cell line HPL1D, derived from a female non-smoker without lung cancer [50], was kindly provided to us by Dr. T. Takahashi (Center of Neurological Diseases and Cancer, Nagoya University Graduate School of Medicine, Nagoya, Japan) and Dr. Hildegard M. Schuller, Department of Pathobiology, College of Veterinary Medicine, University of Tennessee, Knoxville, TN) [51,52]; and the human MCF-7 breast cancer cell line was obtained from ATCC as a positive control for GPER [44]. Both semi-quantitative PCR (Additional file 1: Figure S1) and real time quantitative PCR (Figure 1A) were performed. These results provide the first evidence of GPER expression in human lung adenocarcinoma cell lines.

### Increased expression of GPER in lung cancer lines

To more accurately examine the mRNA levels of GPER, quantitative real time PCR was performed on lung cancer and normal HBEC lines. As shown in Figure 1A and Table 1, the expression level of GPER is higher in most of the lung cancer cell lines compared to HBECs and HPL1D cells. In 12 lung cancer cell lines tested, GPER relative overexpression ranged from 2 to 10 fold compared to normal HBECs (Table 1). The protein levels of GPER in representative lung adenocarcinoma cell lines, HBEC2-KT, HBEC3-KT, HPL1D, and in MCF-7 breast cancer cells were determined by Western blots using three different antibodies obtained from different commercial sources (Figure 1B-D). Each of the antibodies recognized a number of bands that have, in the case of Novus NBP1-31239 and NLS 4271, been demonstrated by using blocking peptides to be specific and are considered to be various glycosylated forms of GPER [53], or to indicate homodimerization or interaction with other proteins in detergent-resistant complexes [54-56]. Surprisingly, the expression of GPER mRNA and protein are not concordant, nor was there concordance between GPER levels with the three antibodies tested in each cell line, although consistency was observed in H1793 cells for MCF-7 and T47D cells with the 2 Novus antibodies, between the NLS4272 and SC-48524 for H23 cells. Nonetheless, these data for the first time demonstrate GPER expression in lung adenocarcinoma cells and normal lung cells and suggest that the levels of GPER expression are, on average, higher in the NSCLC cells compared to the average of the 3 normal lung cell lines. It is possible that the discordant findings between transcript and protein levels result from altered splicing or mutations in the C-terminus of GPER, but this idea requires further investigation. Three SNPs were described in GPER with one resulting in a single Pro16Leu aa change within the coding region [57]. Additionally, it is also possible that specificity/affinity as well as quality of these GPER antibodies may vary between different sources and the impact of glycosylation and other post-transcriptional modifications on antibody interaction is also an issue.

**Table 1:** GPER mRNA fold level change in lung cancer cell lines compared to normal lung epithelial cells

**Cell line**	**Sex of patient**	**Smoker/non-smoker**	**Level of GPR30**		
			**HBEC 3ET as baseline**	**HBEC 2E as baseline**	**HBEC 2KT as baseline**
HBEC 3ET			1.00	0.96	1.74
HBEC 2E			1.04	1.00	1.81
HBEC 2KT			0.57	0.55	1.00
HPL1D	Female	Nonsmoker no lung cancer	4.06	1.19	0.74
A549	Male	Unknown	0.29	0.28	0.5
NCI-H1792	Male	Smoker	5.15	4.96	8.99
NCI-H23	Male	Smoker	1.97	1.89	3.43
NCI-H1299	Male	Smoker	4.67	4.50	8.15
NCI-H2073	Female	Smoker	1.09	1.05	1.90
NCI-H1395	Female	Smoker	4.80	4.63	8.38
NCI-H1944	Female	Smoker	2.56	2.46	4.46
NCI-H1793	Female	Non-Smoker	1.34	1.29	2.34
MCF7	Female	Unknown	9.74	9.38	16.98

### Elevated levels of GPER expression in mouse and human lung cancer tissues

The expression pattern of GPER in normal and lung cancer tissues was examined using Immunohistochemistry (IHC) staining.

**Figure 2:**
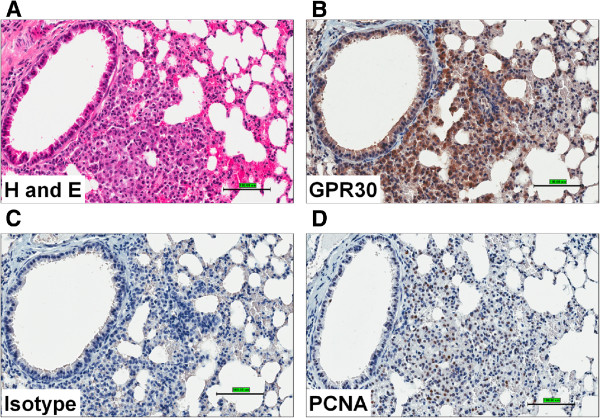
**Expression of GPER/GPR30 in mouse lung tumors. **(**A**) IHC analyses of mouse lung cancer (induced with MCA-BHT). The whole lungs were stained with H & E (**A**), GPR30 C-terminal antibody (Novus Biologicals, 10 μg/ml) (**B**) along with isotype control (**C**) and PCNA (**D**) using standard IHC protocols.

**Figure 3:**
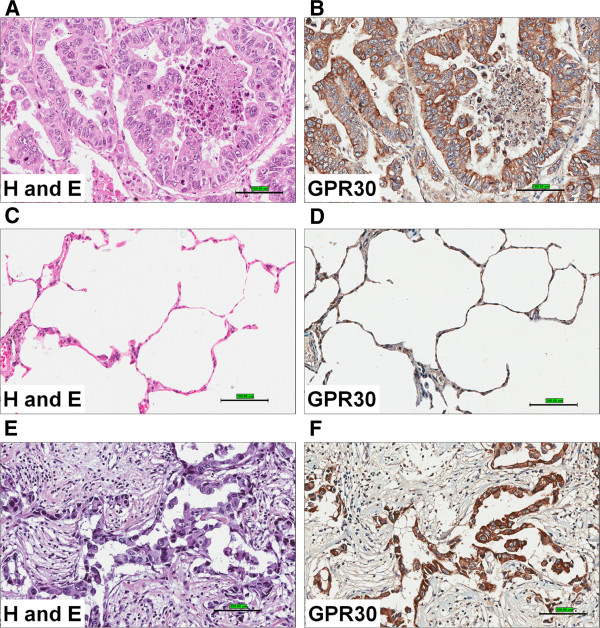
**GPER/GPR30 expression in human lung and breast tumors. **The TMA slides, ‘Multiple lung cancer test array with unmatched normal adjacent tissue’ (US BioMax Inc, #LC242) and ‘human breast cancer test tissue array with self-matching or unmatched normal adjacent tissues’ (US Biomax Inc #BR241) were stained with H&E and anti-GPR30 (10 μg/ml). (A-B). The representative tissue spot/core images of Hand E (**A**) and GPR30 (**B**) staining for lung cancer (adenocarcinoma) are shown. (**C-D**). The representative normal lung tissue for H and E (**C**) and GPR30 staining (**D**) are shown. (**E-F**) The breast cancer TMA slides were stained with H&E and anti-GPR30 as a positive control for GPR30 expression. The images were collected at 200X magnification using Aperio image scope. The scale bars indicate 100 μm.

**Figure 4:**
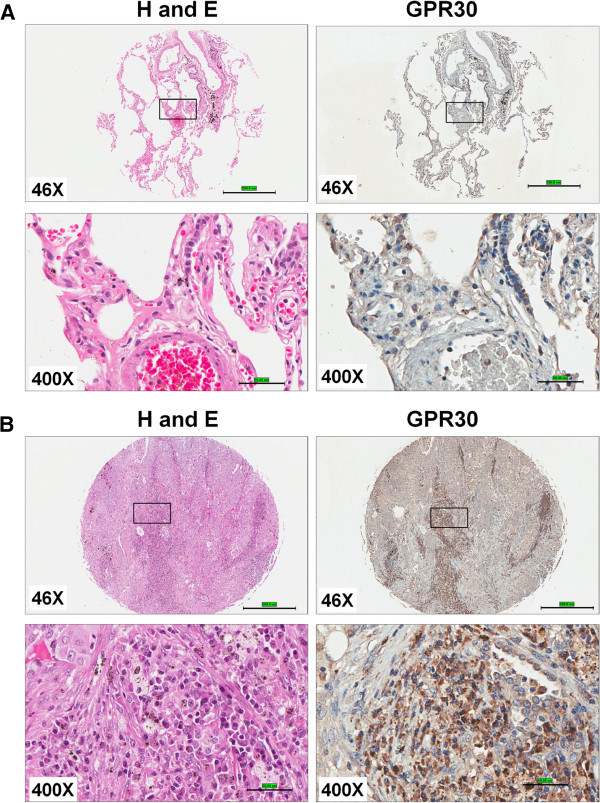
**GPER/GPR30 expression in human lung tumors. **The TMA slides were stained with H&E and anti-GPR30. The representative tissue spots/cores for normal (**A**) and squamous cell carcinoma (**B**) are shown. The left panels represent H and E staining of normal lung (**A**) and squamous cell carcinoma (**B**) and right panels representGPR30 IHC staining at 46X (scale bar 500 μm) and 400X (scale bar 50 μm) magnification The images were collected using Aperio imagescope.

**Figure 5:**
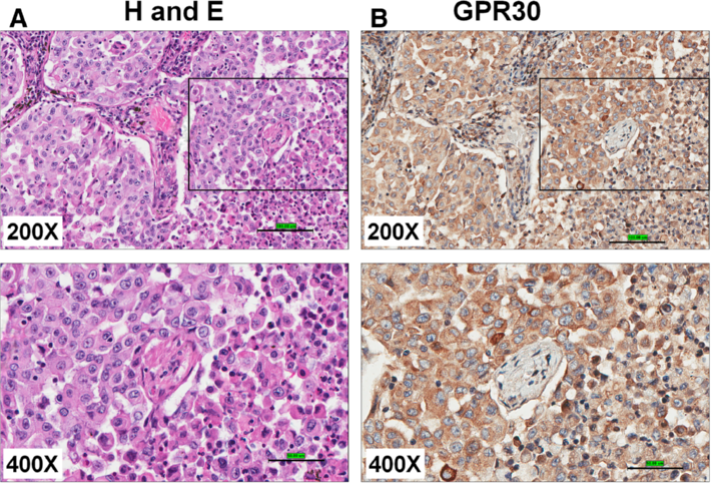
**GPER/GPR30 expression in human large cell lung carcinoma. **The TMA stained with H&E and anti-GPR30. The representative tissue images of stained with H and E (**A**) and anti-GPR30 (**B**) of large cell carcinoma are shown at 200X and 400X magnifications. The scale bar for 200X is 100 μm and 400X is 50 μm. The images were collected using Aperio imagescope.

**Figure 6:**
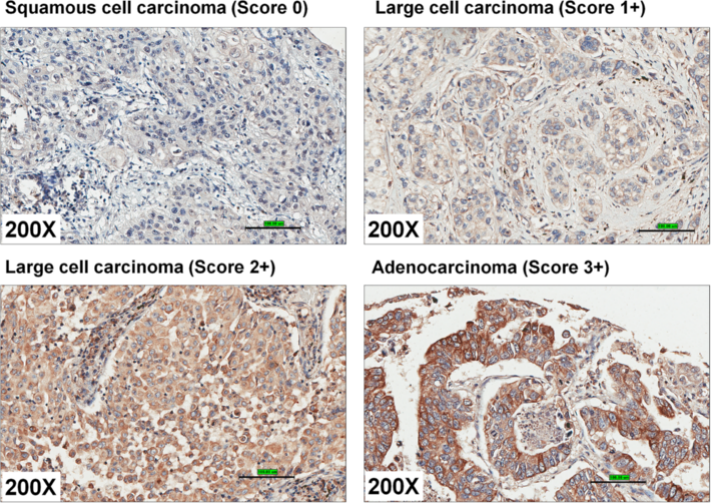
**Scoring of GPER/GPR30 expression in lung tumors. **The scoring pattern for GPR30 staining as follows. Score 0, negative staining for all cells; score 1+, weakly positive for cytosolic staining in <10% of cells; score 2+, moderate to strong positive staining covering between 10 to 50% of cells and score 3+, strongly positive staining including >50% cells. The images are shown at 200X magnification and the scale bar indicates 100μm. The images were collected using Aperio imagescope.

a) *Mouse lung tumors:* The IHC was performed to determine the expression of GPER and proliferating cell nuclear antigen (PCNA) in paraffin embedded tissue sections of mouse lung tumors. The mouse lung tumors were induced using 3-methylcholanthrene-butylated hydroxytoluene (MCA-BHT). The representative H&E stained (Figure 2A) and IHC images of mouse lung tumor sections are shown (Figure 2B-D). The isotype control antibody showed no positive staining (Figure 2C). The expression of GPER and PCNA staining appear to localize to the same regions (Figure 2D) suggesting that GPER is overexpressed in proliferating tumor cells.

b) *Human lung tumors:* The human multiple lung cancer tissue arrays with unmatched normal adjacent tissues (US Biomax Inc #LC242 (10 cases) and LC1005 (77 cases) were used to determine the expression of GPER patterns. A human breast cancer test tissue array was used with self-matching or unmatched normal adjacent tissues (US Biomax Inc #BR241) as a positive control. The representative lung tumor shown in Figure 3A is from male (age 57) and classified as adenocarcinoma, Grade II and malignant tumor. This tissue micro array LC242 represents 10 cases of lung tumor with 2 non-neoplastic tissue (duplicated core in 1.5 mm size per case) sections. The expression of GPER in another TMA (LC1005), containing 77 cases of lung cancer tissues along with normal (8 cases) and cancer adjacent tissues (42 cases) were also analyzed. The representative images of H and E and IHC analysis of GPER expression in different types of lung cancers are shown in Figure 3 A-D along with normal adjacent lung tissues. The positive staining for GPER in human breast cancer (Infiltrating ductal carcinoma not otherwise specified (NOS), Grade II, malignant) is much stronger compared to non-tumor regions of matched adjacent tissues, which served as positive control in these IHC studies (Figure 3E-F). The expression of GPER is also elevated in squamous cell carcinoma and large cell carcinoma (Figures 4 and 5). The overall results suggest that the GPER is overexpressed in lung cancer tissues compared to normal/adjacent lung tissues.

c) *Scoring of GPER staining:* The scoring of the GPER staining was performed by two independent pathologists. The scoring pattern for GPER staining as follows. Score 0, negative staining for all cells; score 1+, weakly positive for cytosolic staining in <10% of cells; score 2+, moderate to strong positive staining covering between 10 to 50% of cells and score 3+, strongly positive staining including >50% cells (Figure 6). All the scoring was done in a blinded manner regarding tumor type/stage data. The comparison between two non-parametric groups was done using Mann–Whitney U test. The GPER scores were compared between the cancer tissues and cancer-adjacent tissue/normal lung tissue. The IHC scores were grouped into two groups, negative or weakly positive (0 and 1+) and moderately to strongly positive (2+ and 3+) (Table 2). IHC quantification (Figure 6 and Table 2) suggests that GPER is significantly overexpressed in lung tumors compared to either normal lung or cancer-adjacent tissues. GPER positive staining (moderate intensity) was observed on the alveolar macrophages in the normal lungs. We did not observe any detectable GPER IHC positive staining in the normal lung epithelium. GPER is significantly overexpressed (2–3 score) in > 80% the adenocarcinomas (p<0.0001), 75% in large cell carcinoma (p<0.0001) and 60% in squamous cell carcinoma (p<0.0001). Overall, > 76% of all lung cancer tissues showed positive GPER staining (score 2 to 3) whereas < 3% of normal lung tissues/adjacent tissues (score 2 to 3) showed any detectable GPER expression. We conclude that GPER expression is increased in lung cancer.

**Table 2:** GPER IHC scoring in tissue microarrays

**Tissue**	**Score 0-1+**	**Score 2-3+**	***P-values *(compared with cancer adjacent tissues)**	***P-values *(compared with normal lung tissues)**
Adenocarcinoma	2 of 15 (13.3%)	13 of 15 (86.7%)	*P<0.0001*	*P=0.0007*
Squamous cell carcinoma	13 of 33 (40%)	20 of 33 (60.0%)	*P<0.0001*	*P= 0.0024*
Large cell carcinoma	2 of 8 (25%)	6 of 8 (75%)	*P<0.0001*	*P=0.0011*
Metastatic adenocarcinoma	1 of 6 (16.7%))	5 of 6 (83.3%)	*P=0.0002*	P=0.0013
Cancer adjacent lung tissue	41 of 42 (97.6%)	1 of 42 (2.4%)		No significant
Normal lung tissue	8 of 8 (100%)	0 of 8 (0)	No significant	

The scoring pattern for GPR30 staining as follows. Score 0, negative staining for all cells; score 1+, weakly positive for cytosolic staining in <10% of cells; score 2+, moderate to strong positive staining covering between 10 to 50% of cells and score 3+, strongly positive staining including >50% cells.

## Discussion

GPER is an E_2_ binding, G-protein coupled membrane receptor [39-42,58] that was reported to be overexpressed in breast [40,59] endometrial [60,61], ovarian [62] and thyroid cancers [63]. The results presented here extend these observations to show that different types of lung cancers including adenocarcinomas, squamous cell carcinoma and large cell carcinomas express higher GPER than normal lung tissue.

Here, we demonstrate for the first time that GPER is overexpressed in lung tumors and lung adenocarcinoma cell lines relative to normal lung and immortalized normal lung cell lines, although the expression of GPER transcript in HPL1D cells is higher than HBECs. GPER has been postulated to be involved in E_2_-activation of EGFR [38]. Filardo’s group showed a link between GPER expression and tumor progression and increased tumor size in breast cancer patients [40]. Recently, GPER overexpression was reported to be independent of ERα expression in breast cancer patient samples, indicating the importance of GPER in ERα negative tumors [64]. GPER and EGFR expression were correlated in endometrial adenocarcinoma [60]. Further, overexpression of GPER in advanced stage endometrial adenocarcinoma correlated with poor survival [60]. Other studies also suggest increased GPER in breast, ovarian and endometrial cancers correlates with disease severity and reduced survival [40,59,60,62,65]. These results are in agreement with studies demonstrating association of GPER overexpression in other cancers [40,59,60,62,64,65], although the scoring patterns and correlation of expression levels to disease state may vary among these studies. A limitation of our study is that the average GPER staining scores among different lung cancer grades (I (10 cases), II (30 cases), III (16 cases)) were not significantly different. One other limitation of the current study is that we cannot conclude at this time whether GPER overexpression is cause or consequence of cancer. It is also possible that overexpression of GPER in lung cancers may reflect a defense mechanism to counteract excessive proliferation. Indeed, a recent report by Krakstad *et al.* showed that loss of GPER in ERα-positive endometrial cancers is associated with poor prognosis [66]. Another study showed that the GPER agonist G-1 inhibited E_2_-induced uterine epithelial cell proliferation in mice by repressing MAPK activation, indicating that GPER effects are tissue specific [67]. Because our studies were performed on commercial TMAs, the results cannot be extrapolated to correlate GPER expression levels to disease outcomes. Clearly, this is a next logical step in light of the novel findings.

We observed no differences in GPER expression between adenocarcinoma cell lines or tumors from male and female patients, similar to the previous findings of no difference in ERα or ERβ expression in NSCLC cells and tumors based on gender [20,68-70]. In Western blots, rather than rely on one GPER antibody in our study, we used 3 different commercial antibodies to determine the correlation between mRNA and protein levels. It is indeed evident from our Western blot data that GPER appears to have different MW forms, likely due to glycosylation [53], dimerization [54,55], and interaction with other membrane proteins [56], and levels in the lung adenocarcinoma cell lines. More trivial explanations for the different staining patterns of GPER in Western blots may be due to differential purity/affinity of the three GPER antibodies as well as their capacity to bind to secondary antibodies. It will be important to determine the nature of these forms by proteomic analysis and gene sequencing to evaluate their biological significance.

The role of GPER as an E_2_ membrane receptor is controversial and its functional significance is unclear. Some reports suggest that GPER is not an estrogen receptor because it does not bind E_2_ and thus still consider it as an orphan GPCR [27,71-73]. The recent identification of estrogen receptor splice variant called ERα36 adds one more layer of complexity to estrogen biology and the role of GPER [72]. ERα36 was reported to be responsible for E_2_ induced non-genomic signaling rather than GPER [72].

Mechanism-based studies showed that GPER transactivates EGFR in breast cancer cells [38,39,74-76] as well as in thyroid, endometrial and ovarian cancer cell lines [61,63,77,78]. Inhibitors of EGFR tyrosine kinase (gefitinib) and ER (fulvestrant, ICI 182,780) were reported to synergize their anti-proliferative effects in NSCLC [19]. Given the importance of EGFR signaling as a therapeutic target in lung cancer [79,80], further examination of the effect of EGF, heregulin, and amphiregulin on GPER expression and function in lung cancer may provide new insights into resistance to EGFR inhibitors and or how estrogens stimulate lung cancer.

## Conclusion

In conclusion, the data presented in this manuscript demonstrate that GPER expression is higher in lung tumors compared to normal lung tissue. While it is not yet clear that elevated GPER expression is a cause of or consequence from lung cancer progression. Functional analysis of the effect of GPER expression will facilitate further delineation of the role of GPER in lung cancer.

## Methods

### Cell lines and mouse lung tissues

Normal human bronchial epithelial cell lines HBEC2-E HBEC2-KT, and HBEC3-KT were kindly provided by Dr. John D. Minna [49]. HPL1D, an SV40-immortalized human small airway epithelial cell line derived from a female non-smoker without lung cancer [50] was kindly provided to us by Dr. T. Takahashi (Center of Neurological Diseases and Cancer, Nagoya University Graduate School of Medicine, Nagoya, Japan) and Dr. Hildegard M. Schuller, Department of Pathobiology, College of Veterinary Medicine, University of Tennessee, Knoxville, TN) [51,52]. Human lung adenocarcinoma cell lines A549, NCI-H1435, NCI-H1395, NCI-H1944, NCI-H1792, NCI-H1793, NCI-H2073, NCI-H23, and NCI-H1299 and human breast cancer cell lines MCF-7 and T47D were purchased from ATCC (Manassas, VA, USA) and used within 10 passages from the time of purchase from ATCC. The growth conditions for each of these lines were described previously [21,22]. Cell culture media supplies obtained either from Invitrogen (Carlsbad, CA, USA) or Mediatech, Inc. (Manassas, VA, USA). All the experimental protocols, usage of human cell lines and chemicals have been approved by the Institutional Biosafety Committee (IBC) at University of Louisville. The mouse lung tumor tissue sections were obtained from MCA-BHT induced lung cancer mouse model (Elangovan *et al* unpublished). All the animal experimental protocols have been approved by the Institutional Animal Care and Use Committee (IACUC) at University of Louisville.

### RNA isolation, cDNA synthesis, RT PCR

Total RNA was isolated using Qiagen RNAasy mini kit (Qiagen, Valencia, CA, USA) according to manufactures’ protocols and as described [21,22]. The isolated total RNA was treated with DNAse followed by synthesis of cDNA by reverse transcriptase (Applied Biosystems, Carlsbad, CA, USA). The similar reaction was also performed without reverse transcriptase as a control. The regular PCR reaction with Mango Taq Polymerase was performed on the above cDNA samples as templates to detect the presence of GPER using specific primers (FP 5^′^ AGTCGG ATGTGAGGTTCAG 3^′^ and RP 5^′^ TCTGTGT GAGGAGTGCAAG 3^′^) for GPER and Human ribosomal phosphor-protein (36B4) as reference marker [76]. The PCR was also performed on the cDNA reaction mix that did not contain reverse transcriptase as a negative control.

### Real time PCR

For quantitative real-time PCR, 1 μg of total RNA was reverse transcribed in 50 μl reaction using TaqMan reverse transcription reagents (Applied Biosystems) using random hexamer primers. 2 μl of cDNA and the 1 μM real time PCR primers were used in a final 20 μl qPCR reaction with ‘power SYBR-green master mix’ (Applied Biosystems). The sequences of the real time primers as follows: hGPER FP: 5^′^ AGTCGGATGTGAGGTTCAG 3^′^; hGPER RP: 5^′^ TCTGTGTGAGGAGTGCAAG 3^′^[81]; h36B4 FP: 5^′^ CTCAACATCTCCCCCTTCTC 3; h36B4 RP: 5^′^ CAAATCCCATATCCTCGTCC 3^′^. Real time qPCR was performed in ABI-Prism 7900 sequence detect system (Applied Biosystems). Expression of the target genes was normalized to ribosomal phosphoprotein (36B4) and displayed as fold change relative to the wild type sample.

### Western blots

The cell lysates were prepared using RIPA plus buffer. 10 μg of total lysates were loaded on to SDS PAGE gels and detected using antibodies anti-GPER antibodies (Novus Biologicals, Littleton, CO, USA), Santa Cruz Biotechnology Inc (Santa Cruz, CA, USA). The membrane was stripped and used for beta-actin detection with anti-beta-actin-HRP antibody (Santa Cruz Biotechnology Inc.). The GPER antibodies obtained from Novus Biologicals were raised against synthetic peptide contain a sequence corresponding to a region within amino acids 244 and 306 (NBP1 31239) and second one against the synthetic peptide [KLH conjugated] made to the C-terminal of human GPER (NLS 4272). Another antibody from Santa Cruz Biotechnology Inc. is raised against internal region of human GPER. β-Actin-HRP antibody obtained from Santa Cruz Biotechnology Inc.

### Immunohistochemistry

The paraffin embedded lung tumor tissue sections were routinely deparaffinized and endogenous peroxidase was quenched with 3% H_2_O_2_ in 1XPBS. The epitope retrieval was performed by heating for 30 min in sodium citrate buffer (pH6.0) in a water bath at 95-100°C. The anti-GPER antibody (Novus Biologicals) and isotype control used as primary antibodies. After 1 hr incubation with the primary antibody at room temperature, the slides were washed twice with 1XPBS (5 min per wash), and then incubated with the secondary antibody solution for 30 min at room temperature. Visualization of GPER positive cells was done by using ABC staining system (Santa Cruz Biotechnology). Negative controls for all staining were done by omitting primary antibodies as well as use of isotype control antibodies. The sections were evaluated by Aperio Imagescope and quantified the number of positive cells at 200x magnification.

### Tissue microarrays

Human lung cancer (LC 242, LC1005) and breast cancer (BR 241) tissue microarrays used in this study were purchased from US Biomax Inc. (Rockville, MD, USA).

### Scoring of GPER expression

The scoring of the GPER staining was performed by Swarupa Gadre, M.D., pathologist, U of L and reconfirmed by an independent pathologist, A. Bennett Jensen, M.D., Brown Cancer Center, U of L. The scoring pattern for GPER staining as follows: Score 0, negative staining for all cells; score 1+, weakly positive for cytosolic staining in <10% of cells; score 2+, moderate to strong positive staining covering between 10 to 50% of cells and score 3+, strongly positive staining including >50% cells. For statistical purposes IHC scores were grouped into two groups, negative or weakly positive (0 and 1+) and moderately to strongly positive (2+ and 3+). All the scoring was done in a blinded manner to tumor type/stage data of tissue microarray. The pairwise comparisons were performed (cancer vs adjacent tissues as well as cancer vs normal lung tissues) using Mann–Whitney U test in Graphpad Prism software.

## Abbreviations

GPR30/GPER: G-Protein coupled estrogen receptor 1; E_2_: 17-β estradiol; ER: Estrogen receptor; ERE: Estrogen responsive element; EGFR: Epidermal growth factor receptor; MAPK: Mitogen activated protein kinase; NSCLC: Non-small cell lung carcinoma; TMA: Tissue micro array.

## Competing interests

The authors declare no competing financial interests.

## Authors’ contributions

Dr. VRJ designed and performed the experiments as well as written the manuscript. Ms. R performed some of the qPCR and western blot experiments. Dr. B participated in design of research and writing of the manuscript. Dr. CMK provided the RNA and lung adenocarcinoma cell line samples, was involved in discussions for the design of experiments, performed calculations on the experiments performed by Ms. R, and contributed to the writing of the manuscript. All authors read and approved the final manuscript.

## Pre-publication history

The pre-publication history for this paper can be accessed here:

http://www.biomedcentral.com/1471-2407/12/624/prepub

## Supplementary Material

Additional file 1 **Figure S1. **The semi-quantitative PCR of GPER (GPR30) in lung adenocarcinoma cells. RNA was isolated from each of the indicated cell lines and the cDNA was prepared as described in methods section. The semi-quantitative was performed using GPR30 primers and human ribosomal phosphoprotein (36B4) as reference as described Methods. Click here for file
