# Supplementary figures and images for: Enhanced expression of G-protein coupled estrogen receptor (GPER/GPR30) in lung cancer
Source: BMC Cancer. 2012 Dec 28;12:624. doi: 10.1186/1471-2407-12-624 (PMC3557142; doi:10.1186/1471-2407-12-624)

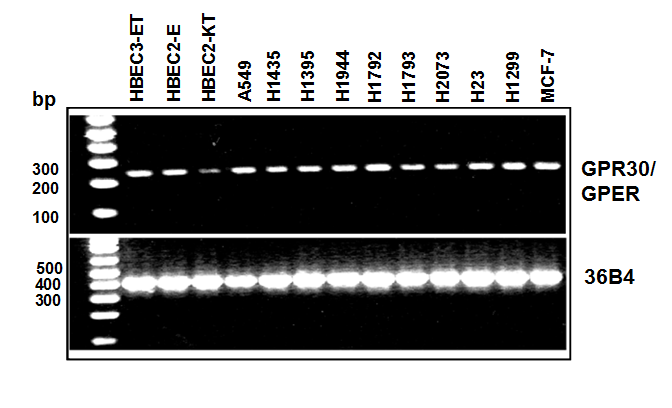

Supplement: Additional file 1 — Figure S1. The semi-quantitative PCR of GPER (GPR30) in lung adenocarcinoma cells. RNA was isolated from each of the indicated cell lines and the cDNA was prepared as described in methods section. The semi-quantitative was performed using GPR30 primers and human ribosomal phosphoprotein (36B4) as reference as described Methods. [file 1471-2407-12-624-S1.tiff]
